# Supplementary material for: The Zebra Mussel (Dreissena polymorpha) as a Model Organism for Ecotoxicological Studies: A Prior 1H NMR Spectrum Interpretation of a Whole Body Extract for Metabolism Monitoring
Source: Metabolites. 2020 Jun 18;10(6):256. doi: 10.3390/metabo10060256 (PMC7345047; doi:10.3390/metabo10060256)
Supplement: Supplementary file 1 [file metabolites-10-00256-s001.zip › Supporting information_rev.pdf]

Supporting informations

The zebra mussel (*Dreissena polymorpha*) as a model organism for ecotoxicological studies: a prior ^{1}H NMR spectrum interpretation of a whole body extract for metabolism monitoring.

Sophie Martine Prud'homme [1, 2*], Younes Mohamed Ismail Hani [1], Neil Cox[3], Guy Lippens[3], Jean-Marc Nuzillard[4] and Alain Geffard[1,*]

[1] Université de Reims Champagne-Ardenne, UMR-I 02 SEBIO (Stress Environnementaux et Biosurveillance des milieux aquatiques), Reims, France
[2] Université de Lorraine, CNRS, LIEC, F-57000, Metz, France (Curent affiliation)
[3] TBI, Université de Toulouse, CNRS, INRA, INSA de Toulouse, 135 avenue de Rangueil, 31077 Toulouse CEDEX 04, France
[4] Université de Reims Champagne Ardenne, CNRS, ICMR UMR 7312, 51097 Reims, France

* Correspondence: sophie.prud-homme@univ-lorraine.fr, alain.geffard@univ-reims.fr

Summary of supporting information

Supplementary Tables:

Table S1: Page S2
Table S2: Page S2
Table S3: Page S2
Table S4: Page S3
Table S5: Page S4
Table S6: Page S5

Supplementary Figures:

Figure S1: Page S6
Figure S2: Page S6

Supplementary Table 1 – Physicochemical parameters of caging sites. Data are expressed as mean values ± SD during the 2 months of caging. dO2: dissolved oxygen.

	Conductivity (µS/cm)	dO2 (mg/L)	pH	Temperature (°C)
**Saint-Mihiel**	642.67 ± 68.69	11.07 ± 0.76	8.14 ± 0.28	9.58 ± 2.20
**Lumes**	551.20 ± 70.38	9.69 ± 1.11	8.04 ± 0.19	9.85 ± 2.39
**Nouzonville**	516.20 ± 103.94	9.72 ± 1.26	8.34 ± 0.25	9.84 ± 2.37
**Liverdun**	369.60 ± 37.55	10.25 ± 1.01	8.11 ± 0.21	10.07 ± 2.79

Supplementary Table 2 – Heavy metal (Cd, Cu, Ni, and Zn) concentration in soft tissues of zebra mussels from "Lac du Der-Chantecoq" population before (Control T0) and after 2 months caging in Saint-Mihiel, Lumes, Nouzonville or Liverdun sites. Soft tissues were mineralized with Suprapur® nitric acid for 24 h at 80 °C. The resulting acidic solutions were adjusted to 10 mL with ultrapure water, and samples were analyzed using an inductively coupled plasma optical emission spectrometer (ICP-AOS, Thermo Scientific iCAP 6300 DUO). Results are expressed as mean µg/kg wet weight ± SD of 3 pools of 1.5g of total body weight (10 to 16 individuals).

	Cd (µg/kg)	Ni (µg/kg)	Cu (µg/kg)	Pb (µg/kg)	Zn (µg/kg)
**Control T0**	0,05 ± 0.01	0,65 ± 60.0	2,18 ± 0.72	0,03 ± 0.00	10,91 ± 0.68
**Saint-Mihiel**	0,06 ± 0.01	0,71 ± 0.25	2,73 ± 0.80	0,06 ± 0.01	9,50 ± 1.09
**Lumes**	0,07 ± 0.02	0,64 ± 0.02	2,08 ± 0.63	0,13 ± 0.09	10,07 ± 2.49
**Nouzonville**	0,07 ± 0.01	0,87 ± 0.13	2,13 ± 0.23	0,07 ± 0.01	10,11 ± 0.56
**Liverdun**	0,06 ± 0.01	0,56 ± 0.09	3,17 ± 0.59	0,14 ± 0.05	11,40 ± 2.50

Supplementary Table 3 – Organic pollutants concentration in soft tissues of zebra mussels from "Lac du Der-Chantecoq" population before (Control T0) and after 2 months caging in Saint-Mihiel, Lumes, Nouzonville or Liverdun sites. Freeze-dried homogenized mussels were treated with a modified QuEChERS extraction approach [1] and analyzed by atmospheric pressure gas chromatography/spectrometry (APGC), using an Agilent 7890B GC system (Agilent, Palo Alto, CA, USA). Results are expressed as mean µg/kg dry weight of 3 pools of 3g of total body weight (20 to 30 individuals). Σ HAPs: sum of all Polycyclic Aromatic Hydrocabones. Σ PBDEs: sum of all Polybrominated Diphenyl Ethers. Σ PCBs: sum of all PolyChlorinated Biphenyls.

	Σ PAHs (µg/kg)	Σ PBDEs (µg/kg)	Σ PCBs (µg/kg)
**Control T0**	0,00	0,00	0,00
**Saint-Mihiel**	0,00	0,00	0,00
**Lumes**	1,23	0,12	0,00
**Nouzonville**	1,05	0,13	0,00
**Liverdun**	0,00	0,85	0,15

[1] Kalachova, K.; Pulkrabova, J.; Drabova, L.; Cajka, T.; Kocourek, V.; Hajslova, J. Simplified and rapid determination of polychlorinated biphenyls, polybrominated diphenyl ethers, and polycyclic aromatic hydrocarbons in fish and shrimps integrated into a single method. Anal. Chim. Acta 2011, 707, 84–91, doi:10.1016/j.aca.2011.09.016.

Supplementary Table 4 – Acquisition and post-processing parameters of 1D and 2D NMR spectra acquired on a 600 MHz spectrometer. *Spectra acquisition was performed on a Bruker AVANCE III 600 MHz spectrometer equipped with a z-gradient 5-mm TCI cryoprobe at the ICMR (Institute of Molecular Chemistry) of Reims Champagne Ardennes university, France.*

	^{1}H	^{1}H-^{1}H JRES	^{1}H-^{1}H COSY	^{1}H-^{1}H TOCSY	^{1}H-^{13}C HSQC
**AQUISITION**					
Acquisition sequence	noesygppr1d	jresgpprqf	cosygpprqf	dipsi2gpphpr	hsqcedetgpsisp2.2
Spectrometer frequency (MHz)	600.16	[F1;F2]: 600.16	[F1;F2]: 600.16	[F1;F2]: 600.16	[F1]: 150.91<br>[F2]: 600.16
Acquisition mode (QF)	DQD	DQD	DQD	DQD	DQD
Receiver gain (RG)	287	575	645	645	2050
Dwell time (µsec)	41.6	83.2	83.2	83,2	83.2
Pre-scan delay (DE) (µsec)	10	10	10	10	10
Nb dummy scan (DS)	4	8	8	128	256
Nb of Scans (NS)	128	32	4	8	8
Acquisition time F1 (sec)	2.73	1.60	0.26	0.06	0.04
Acquisition time F2 (sec)	-	0.68	0.34	0.34	0.13
Size of FID F1 (TD)	65536	64	1536	768	2048
Size of FID F2 (TD)	-	8192	4096	4096	1514
Spectral width F1	20 ppm	10 ppm	10 ppm	10 ppm	10 ppm
Spectral width F2	-	40 Hz	10 ppm	10 ppm	184 ppm
**POST-PROCESSING**					
Real data point F1	128 K	128	2048	2048	1024
Real data point F2	-	8192	4096	4096	2048

Supplementary Table 5 – Acquisition and post-processing parameters of 1D and 2D NMR spectra acquired on a 800 MHz spectrometer. *Spectra acquisition was performed on a Bruker Avance III HD 800 MHz spectrometer equipped with a 5 mm quadruple resonance QCI-P (H/P–C/N/D) cryogenically cooled probe head at the MetaToul.*

	**^{1}H-^{13}C HSQC**	**^{1}H-^{31}P HSQC TOCSY**
**AQUISITION**		
Acquisition sequence	hsqcetgpsisp2.4	na_hsqcetf3gpxy
Spectrometer frequency (MHz)	[F1]: 201.20<br>[F2]: 800.13	[F1]: 323.90<br>[F2]: 800.13
Acquisition mode (QF)	DQD	DQD
Receiver gain (RG)	912	32
Dwell time (µsec)	44.8	44.8
Pre-scan delay (DE) (µsec)	10	10
Nb dummy scan (DS)	16	16
Nb of Scans (NS)	64	64
Acquisition time F2 (sec)	0.11	0.11
Acquisition time F1 (sec)	0.04	0.05
Size of FID F2 (TD)	2048	2048
Size of FID F1 (TD)	256	1024
Spectral width F1	184 ppm	30 ppm
Spectral width F2	12 ppm	12 ppm
**POST-PROCESSING**		
Real data point F1	1024	2048
Real data point F2	4096	4096

Supplementary Table 6 – List of the 24 metabolites validated by spiking into Mix samples

Metabolite	Detection in the representative spectra	Spiked concentration (μM)
**Amino acids**		
Asparagine	Yes	52
Glutamine	Yes	36
Glycine	Yes	35
Histidine	Yes	48
Phenylalanine	Yes	306
Tryptophane	Yes	52
Lysine	Yes	94
**Amine compounds & osmolites**		
Choline	Yes	52
Sarcosine	No	50
**Nucleotides**		
ATP	Yes	55
ADP	Yes	48
AMP	Yes	47
UTP	No	49
UDP	No	51
UMP	Yes	26
IMP	No	46
Adenosine	Yes	50
**Coenzymes**		
NAD	Yes	52
NADH	Yes	50
NADPH	No	52
**Carbohydrates**		
Glucose	Yes	31
Maltose	Yes	61
Glucose-1-phosphate	No	50
Glucose-6-phosphate	No	50

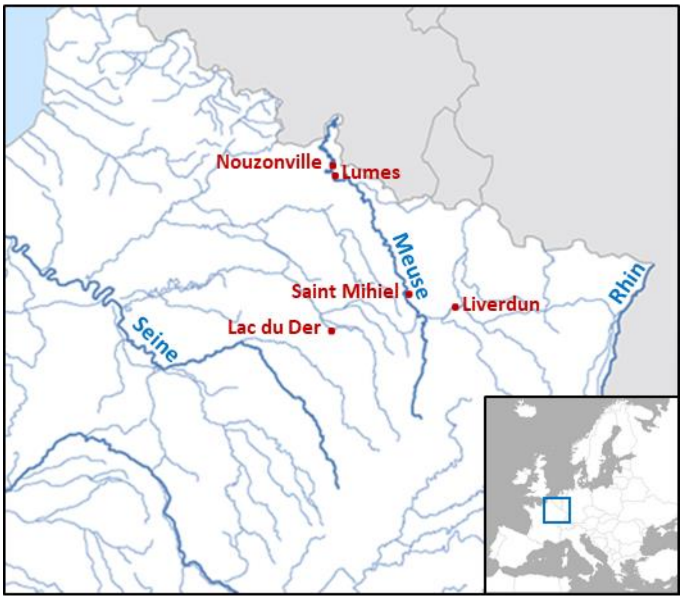

Supplementary Figure 1 - Geographical localizations of the sampling (Lac du Der-Chantecoq; 4°45'00" E; 48°34'00" N) and caging sites along the Moselle (Liverdun; 6°4'0.4303" E; 48°44'44.3620" N) and Meuse river (5°32'27.6180" E; 48°52'12.9349" N - 4°44'28.1306" E; 49°48'58.8740" N - 4°46'29.5799" E; 49°44'18.4070" N). All sites are located in France.

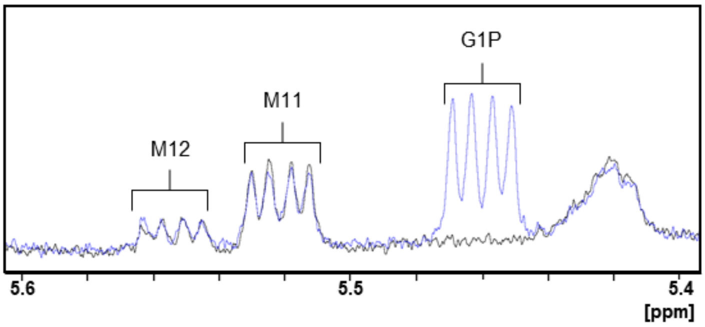

Supplementary Figure 2 – 5.4 to 5.6 ppm region of the *D. polymorpha* ^{1}H representative spectrum. Pairs of doublets characteristic of sugar 1-phosphate resonance from G1P (glucose 1-phosphate), unknown metabolite M11 (peaks 178 & 179) and unknown metabolite M12 (peaks 180 & 181) are indicated by brackets. The black spectrum corresponds to original Mix sample spectrum, and the blue one to Mix sample spiked with glucose 1-phosphate.
